# Genome-wide characterization and sequence polymorphism analyses of cysteine-rich poly comb-like protein in *Glycine max*

**DOI:** 10.3389/fpls.2022.996265

**Published:** 2022-09-20

**Authors:** Tayyaba Nisar, Muhammad Hammad Nadeem Tahir, Shahid Iqbal, Muhammad Sajjad, Muhammad Azhar Nadeem, Ghulam Qanmber, Ayesha Baig, Zulqurnain Khan, Zhengyun Zhao, Zhide Geng, Shoaib Ur Rehman

**Affiliations:** ^1^Institute of Plant Breeding and Biotechnology, Muhammad Nawaz Shareef (MNS) University of Agriculture, Multan, Pakistan; ^2^Department of Biosciences, Commission on Science and Technology for Sustainable Development in the South (COMSATS) University Islamabad, Islamabad, Pakistan; ^3^Faculty of Agricultural Sciences and Technologies, Sivas University of Science and Technology, Sivas, Turkey; ^4^State Key Laboratory of Cotton Biology, Cotton Research Institute of Chinese Academy of Agricultural Sciences, Anyang, China; ^5^Department of Biotechnology, Commission on Science and Technology for Sustainable Development in the South (COMSATS), University Islamabad, Abbottabad Campus, Abbottabad, Pakistan; ^6^Institute of Food Crops, Yunnan Academy of Agricultural Sciences, Kunming, China

**Keywords:** soybean, phylogenetic analyses, kompetitive allele specific PCR, association analyses, drought, *GmCPP*

## Abstract

Cysteine-rich poly comb-like protein (*CPP*) is a member of cysteine-rich transcription factors that regulates plant growth and development. In the present work, we characterized twelve *CPP* transcription factors encoding genes in soybean (*Glycine max*). Phylogenetic analyses classified *CPP* genes into six clades. Sequence logos analyses between *G. max* and *G. soja* amino acid residues exhibited high conservation. The presence of growth and stress-related *cis*-acting elements in the upstream regions of *GmCPPs* highlight their role in plant development and tolerance against abiotic stress. *Ka/Ks* levels showed that *GmCPPs* experienced limited selection pressure with limited functional divergence arising from segmental or whole genome duplication events. By using the PAN-genome of soybean, a single nucleotide polymorphism was identified in *GmCPP-6*. To perform high throughput genotyping, a kompetitive allele-specific PCR (KASP) marker was developed. Association analyses indicated that *GmCPP-6-T* allele of *GmCPP-6* (in exon region) was associated with higher thousand seed weight under both water regimes (well-water and water-limited). Taken together, these results provide vital information to further decipher the biological functions of *CPP* genes in soybean molecular breeding.

## Introduction

Identification and genome-wide characterization of plant transcription factors (TFs) bear vital significance (Qanmber et al., 2019). In plants, TFs play a central role in various developmental processes as well as a stress response (Green et al., 1987).

Cysteine-rich polycomb-like proteins (*CPP*-like) belong to a small TFs family characterized by the presence of one or two similar Cys-rich domains known as the CXC domain (also known as the CRC domain), and the TCR motif (Cvitanich et al., 2000; Hauser et al., 2000; Sijacic et al., 2011). Plants and animals contain members of this family, but prokaryotes, yeasts, and fungi lack them. CXC domains of *CPP*-like proteins are highly conserved in different genera and species (Song et al., 2000; Andersen et al., 2007). *CPP*-like genes are involved in plant development, and in cell division control. *CPP* TFs is a small gene family that includes tesmin/*TSO1*-like CXC (TCX) proteins (Andersen et al., 2007). In many plant species, *CPP* TFs have been discovered to have a variety of functions. *TSO1*, the first *CPP* TFs, were identified and characterized in *Arabidopsis thaliana* using map-based cloning, and its biological functions were explored through mutant screening (Riechmann et al., 2000). *TSO1* gene is mainly expressed in flowers, in developing ovules and microspores. The *tso1* mutants show deficiencies in karyokinesis and cytokinesis, as well as a loss of control over directional cellular expansion and coordination of adjacent cell growth (Riechmann et al., 2000; Sijacic et al., 2011).

Many *CPP* genes have been identified in various plant species, including *A. thaliana* (Hauser et al., 1998), *Oryza sativa* (Yang Z. et al., 2008), *Zea mays* (Song et al., 2016b), and *Glycine max* (Zhang et al., 2015). Cucumber (*Cucumis sativus*) plant is susceptible to abiotic stresses due to its high transpiration rate (Zhou et al., 2017). Gene expression of *CsCPP* genes is upregulated in response to abiotic stresses like salt, cold, drought, and ABA, suggesting that *CsCPPs* may play a role in abiotic stress responses (Yang et al., 2019).

Soybean (*G. max* L.) belongs to the leguminous family and is a prominent source of edible oil and is cultivated in different parts of the world (Fehr and Caviness, 1977; Song et al., 2016a). Soybean seed contains 35% protein and 18% oil contents (Wilson, 2004). Abiotic stress factors are major limiting elements affecting its yield and quality. The role of *CPP*-like protein has been reported in the growth and development of *A. thaliana*, *O. sativa*, *Z. mays*, and *C. sativus*. Although *CPP*-like genes have been identified in soybean, more work is required to further decipher their function in *G. max.* The availability of the soybean pan-genome is expected to pave the way for molecular breeding in soybean (Schmutz et al., 2010). Although molecular markers are available, their deployment in soybean molecular breeding remains limited because of cost ineffectiveness while exploring large populations. Kompetitive Allele Specific PCR (KASP), is a high-throughput and breeder-friendly genotyping platform (Neelam et al., 2013). KASP offers cost-effective genotyping by eliminating the need for post-PCR handling (Majeed et al., 2018).

In this study, we characterized twelve *GmCPP* genes and performed systematic analyses using genome-wide structure depiction and sequence polymorphism investigations. We analyzed *GmCPPs* to explore evolutionary relationships, gene structure, conserved motifs, gene duplication, and association of sequence polymorphism with the studied soybean phenotypic traits under well-water (WW) and water limited (WL) conditions. The present work will assist to underpin the evolution of *GmCPPs* and provide information on *GmCPP* genes to be used in soybean molecular breeding.

## Materials and methods

### Sequence identification

The *CPP* gene and encoded proteins in various species like *G. max, O. sativa*, *Z. mays*, *A. thaliana*, *Brassica rapa, G. soja, Cajanus Cajan*, *Chlamydomonas reinhardtii*, *and Selaginella moellendorffi* were downloaded from plant transcription database.^1^ To confirm the retrieved CPP proteins, local BLASTp, NCBI Batch CD-search, Interproscan V. 63^2^ and SMART^3^ were also used. Non-redundant gene members were selected and the rest were excluded for further analyses. Other biophysical characteristics i.e., protein length, molecular weight (MW), isoelectric point (pI), and gravity values for *GmCPP*s were extracted using ExPASy Protparam Tool.^4^ Furthermore, sub-cellular localization of *GmCPPs* was also identified using the Softberry^5^ and CELLO V2.5 web-tool.^6^

### Sequence alignment and evolutionary analysis

Full-length amino acid sequences of all studied species were aligned and two phylogenetic trees were generated using MEGA X using the maximum likelihood method (ML) following parameters as reported by Kumar et al. (2018). The bootstrap method (1,000 replications) was used to determine the dependability of clades. Graphical representation of multiple sequence alignment of conserved CPP amino acid residues in *G. max* and *G. soja* was performed separately by the Clustal W program (Tamura et al., 2011) and WEBLOG webtool.^7^

### Gene structure, protein motif, and *cis*-element analyses

To explore exon/intron structure, bed-files from databases were obtained and analyzed using GSDS 2.0.^8^ Protein motif distributions were determined using the online MEME tool.^9^ For *cis-*element analyses, ∼2 kb upstream regions were analyzed in the PlantCARE database (Lescot et al., 2002) and the elements were characterized on the basis of their predicted biological functions, and graphical representation was done by using TBtool software.

### Gene duplication and synteny analysis

To determine the chromosomal distribution of *GmCPPs*, extracted gff3-files of soybean genome annotation were downloaded from SoyBase (SoyBase.org). Gene duplication analyses were performed following the methods as reported previously (Yang et al., 2017). CIRCOS was used to create the figure and *Ka/Ks* values were calculated using PAL2NAL (Suyama et al., 2006; Krzywinski et al., 2009).

### Soybean plant material and phenotyping

A set of 46 soybean accessions were planted at MNS University of Agriculture, Multan in the springs of 2021 (2021-UAM). Field experiments were carried out under WL and WW experimental units following Augmented design (Check = UAMSB200). The WL experimental units were subjected to drought especially at the flowering stage, whereas, WW experimental units were irrigated after every fortnight (depending upon water requirement). Each soybean genotype was planted on two beds (length × width = 15 × 2.5 ft) on both sides. Plant-to-plant distance was maintained at a distance of 1 ft. Phenotypic data were recorded for plant height, thousand seed weight, pods^–1^ plant, seeds^–1^ pod, seed weight^–1^ pod, seed length, seed thickness, seed width, and pod length from both water regimes.

### Development of single nucleotide polymorphism based kompetitive allele-specific polymerase chain reaction markers for *GmCPP*

Genomic DNA of the investigated soybean germplasm was extracted from young seedling leaves using the CTAB method (Lecharny et al., 2003). The quality of extracted DNA was initially checked by using NANO-Drop (K5800C Micro-Spectrophotometer) followed by running the extracted DNA on 1.0% agarose gel.

The whole genome sequence of three cultivars of soybean (Williams-82, Lee, and Zhonghuang-13) was downloaded from SoyBase.^10^ Local BLAST was performed to identify the sequences of *GmCPPs* in the aforementioned soybean genotypes. For the identification of sequence polymorphism, multiple sequence alignment was performed using the Seqman program in the DNASTAR Lasergene package. Standard kompetitive allele-specific PCR (KASP) guidelines^11^ were followed for the development of KASP primers on the identified single nucleotide polymorphism (SNP) of *GmCPP6*. Allele-specific primers were developed having standard HEX and FAM tails with a targeted SNP at three prime ends. Two reverse primers (allele-specific) and one common forward primer were designed so that the total fragment length was less than 100 bp. The standard KASP reaction mixture, KASP assay, and PCR conditions were followed as reported by Rasheed et al. (2016), Majeed et al. (2018), Ur Rehman et al. (2019, 2021), and Irshad et al. (2019, 2021).

### Statistical analyses

Phenotypic data were analyzed with XLSTAT Software 2014. Student’s *t-*test at *p* less than 0.05 was used to check the effect of each allelic variation on the recorded phenotypic traits.

## Results

### Identification of cysteine-rich polycomb-like protein gene family members in different species

We identified a total of 81 *CPP* genes in nine investigated species including chlorophytes (*C. reinhardtii*), lycophytes (*S. moellendorffii*), *Brassicaceae* (*A. thaliana* and *B. rapa*), *Fabaceae* (*G. max, G. soja*, and *C. cajan*), and *Poaceae* (*Z. mays* and *O. sativa*). Among these, 12 *CPP* genes were shortlisted in *G. max*, 10 in *G. soja*, 16 in *B. rapa*, 11 each in *Z. mays* and *O. sativa*, eight in *A. thaliana*, six in *C. cajan*, four in *S. meollendorffii*, and three in *C. reinhardtii*. A higher number of *CPPs* were identified in *G. max* as compared to chlorophytes and lycophytes indicating a duplication effect on *GmCPPs* in *G. max*. These findings also signify that *CPPs* experienced extension in higher plants. The transcription factor ID, taxonomic ID, and predict sub-cellular localization are presented in Supplementary Table 1. These results showed that the *GmCPP* coding sequence ranged from 1,656 to 2,715 bp for *GmCPP-6* and *GmCPP-11*, respectively. Similarly, an amino acid number of *GmCPP* genes ranged from 483 to 904 for *GmCPP-5* and *GmCPP-11*, respectively. Molecular weight ranged from 54,034.67 to 98,476.32 kDa for *GmCPP12* and *GmCPP-4*, respectively. The isoelectric point of *GmCPP4* was the highest (9.2) and that of *GmCPP-1* was the lowest (5.41). The grand averages of hydropathicity values of all *GmCPPs* were less than zero and ranged from -0.734 for *GmCPP-11* to -0.511 for *GmCPP-4*. In addition, all *GmCPPs* are localized in the nucleus, except *GmCPP-1* and 10.

### Phylogenetic analyses of cysteine-rich polycomb-like protein gene family

**FIGURE 1:**
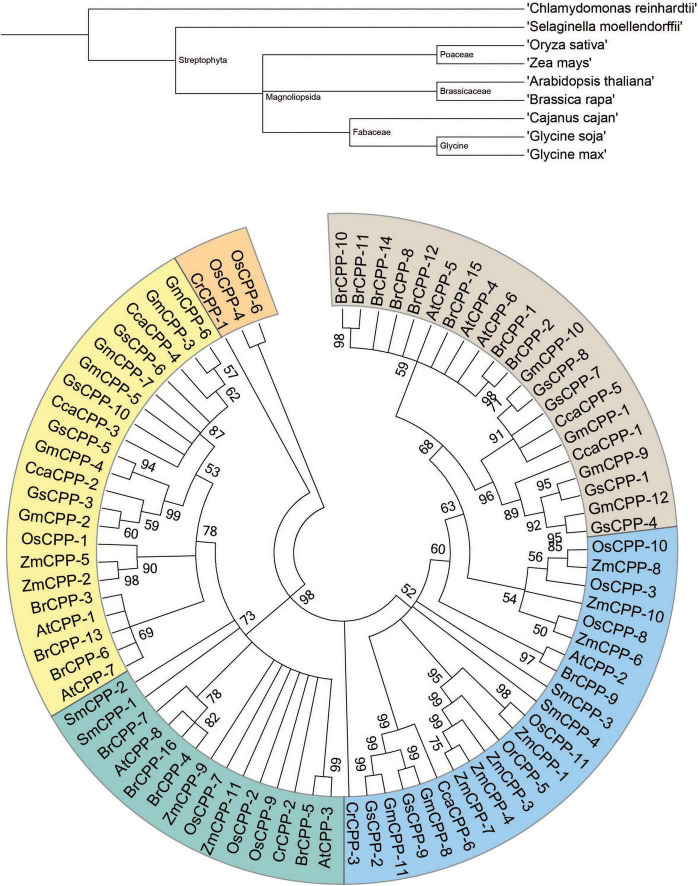
Phylogenetic tree of *GmCPPs* from nine different species. Phylostartum analyses of *CPP* gene family **(Upper portion)**. Phylogenetic and evolutionary relationship of *CPP* gene family in soybean and other plant species **(Lower portion)**.

The phylostratum analyses of *CPP* genes identified the primitive lineage as *CPP* genes, which were also identified in chlorophyte (*C. reinhardtii*) (Figure 1). Further, the *CPP* genes were identified in lychophytes, dicots, and monocots. These outcomes signified that *CPPs* originated from early plants phylostratum and possible orthologs are present throughout kingdom Plantae. An evolutionary tree was generated to determine the phylogenetic relationship among the studied *CPPs*. To indicate the *CPPs* from *C. reinhardtii*, *S. moellendorffii*, *A. thaliana*, *B. rapa*, *G. max, G. soja*, *C. cajan, Z. mays*, and *O. sativa* the prefixes Cr, Sm, At, Br, Gm, Gs, Cc, Zm, and Os were used correspondingly. The phylogenetic analyses divided 81 genes into six clades based on sequence similarities (Figure 1). Clade-I comprised 16 members, Clade-II possessed 13 members, Clade-III contained 14 members, Clade-IV, and V had 15 members each, and Clade-VI contained eight members. Clade-I lacks genes from chlorophytes which suggested the evolution of *CPP* genes after the split of chlorophyte. Interestingly, *CPP* genes from monocot and dicot species were unsystematically distributed to all clades. Further, phylogenetic analyses indicated that *G. max* and *B. rapa* experienced gene family expansion since both have more *CPP* genes compared to other studied organisms.

**FIGURE 2:**
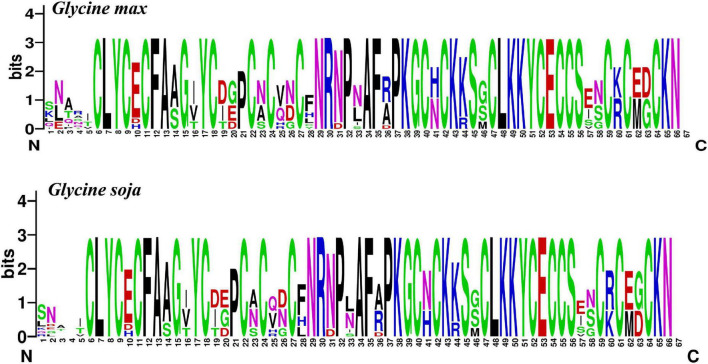
Sequence logo of GmCPP and GsCPP. Amino acid residues shared by two plant species are highly conserved. Each black letter showed the conserved amino acids at a given location.

To explore the conservation of each amino acid residue in GmCPP and GsCPP, multiple sequence alignment was executed to generate sequence logos in *G. max* and *G. soja*. The outcomes showed that the amino acid residue distribution was highly similar at most of the loci among the *G. max* and *G. soja*. For example, some amino acid residues such as C [6], L [7], Y [8], C [9], C [11], F [12], A [13], N [29], A [34], and so on were found to be highly conserved (Figure 2). Phylogenetic analyses also highlight that *GmCPP* and *GsCPP* members lie in close proximity to the evolutionary tree.

### Gene structure, protein motif, and *cis*-acting element analysis

**FIGURE 3:**
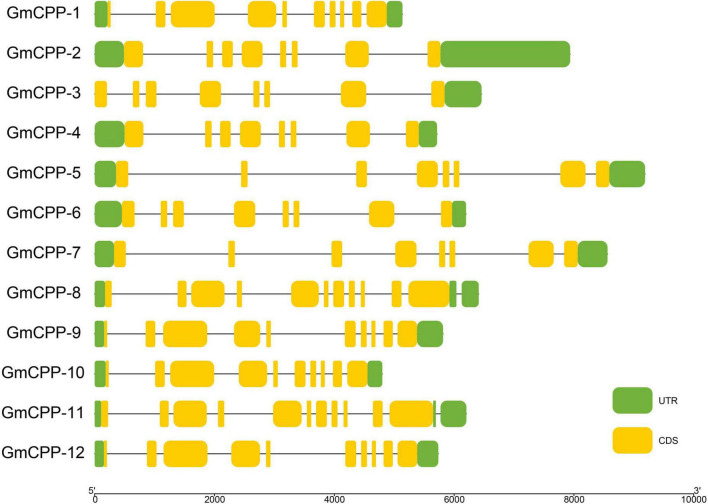
Gene structure display of *GmCPP*. Exon/intron structure display of *GmCPP* genes; Green color shows the upstream and downstream region, yellow color shows exons, black lines show introns.

**FIGURE 4:**
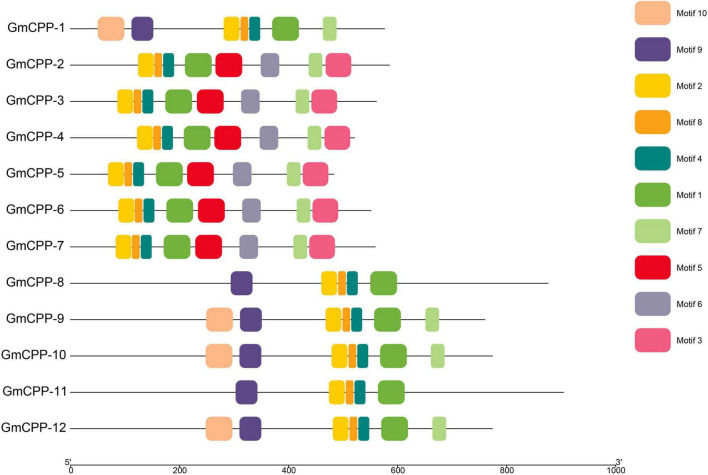
Motifs in all *GmCPP* genes. Distribution of conserved motifs in *GmCPP* are presented in different colors.

It has been well-documented that intron-exon distribution arrangement in a gene is related to its biological function. The intron number in *GmCPP* ranged from 7 to 9 (Figure 3). Conserved domains in each sequence were identified using the CDD tool of NCBI.^12^ All members of the *GmCPP* gene family contain the TCR domain (Supplementary Figure 1). MEME tool was used to explore the conserved motif distributions of *GmCPPs*. The outcomes indicated that most of the *GmCPP* proteins exhibited similar motif distribution patterns such as motifs one, two, and eight exist in almost all proteins (Figure 4). We also identified *cis*-acting elements in the upstream regions of *GmCPPs* and grouped them on the basis of their functional relevance. All *GmCPPs* had *cis*-acting elements related to plant development, stress, and light responses (Supplementary Table 2).

### Chromosomal distribution, gene duplication, and synteny analyses

**FIGURE 5:**
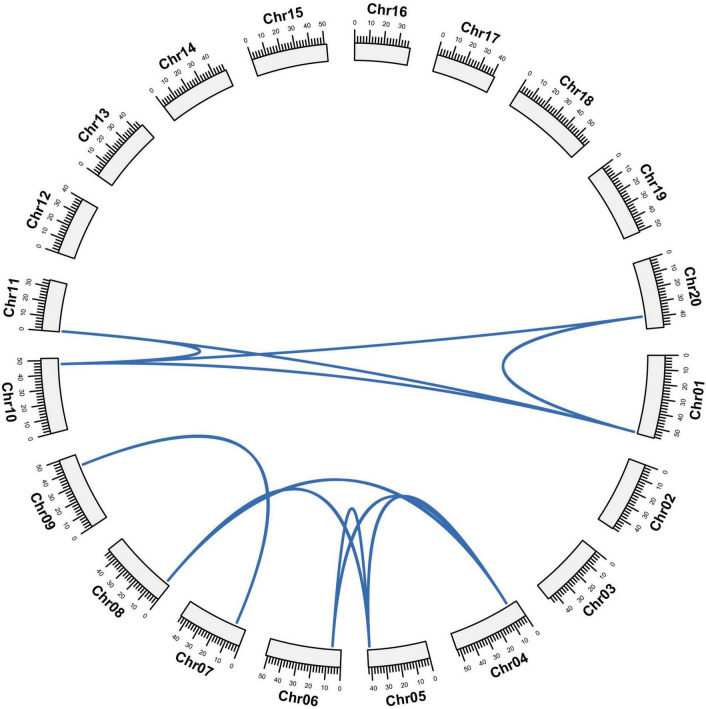
Gene duplication and synteny analysis. Gene duplication among *GmCPP* genes. Blue line shows orthologous/paralogous pair. Gm01 to Gm20 shows the chromosomes of *Glycine max*.

The 12 *GmCPP* genes are scattered on five chromosomes, including three of each gene on chromosomes one and four, respectively. Two each gene are on chromosomes five and 10 while one gene is present on chromosome seven (Supplementary Table 3). To investigate the relationship of gene pairs, we explored the gene locus on a chromosome and executed synteny analyses. Their, synteny analyses showed that *GmCPP* genes were highly conserved among five chromosomes (Figure 5). Whole genome duplication, segmental duplication, and tandem duplication play a vital role in the extension of a gene family (Yang et al., 2017). To investigate the expansion of the *GmCPP* family in soybean, we executed gene duplication analyses in the soybean genome (Supplementary Table 3). Out of all studied gene pairs, 10 gene pairs were attributed to segmental duplication. We also explored the non-synonymous divergence (*Ka*) versus synonymous (*Ks*) values for the *GmCPP* gene pairs. It was found that nine duplicated gene pairs showed *Ka/Ks* values <0.5, whereas, two duplicated gene pairs showed *Ka/Ks* values between 0.5 and 1.0 (Supplementary Table 3). Generally, *Ka/Ks* of the studied gene pairs were <1, showing that the *GmCPP* gene family experienced purifying selection pressure with restricted functional differences.

### Marker trait association analysis

**FIGURE 6:**
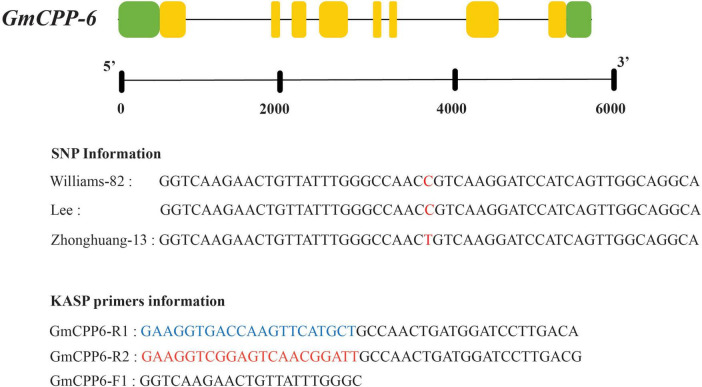
Gene structure and KASP marker development for *GmCPP-6*. Red colored alphabets indicated sequence polymorphism. Nucleotides color blue indicating FAM where nucleotides colored red indicating HEX.

For *GmCPP-6*, a polymorphic site was identified in the coding region. KASP marker was developed at the SNP site. KASP assay results showed that soybean accession having HEX tail has “C” allele while accession having FAM tail has “T” type allele (Figure 6).

**FIGURE 7:**
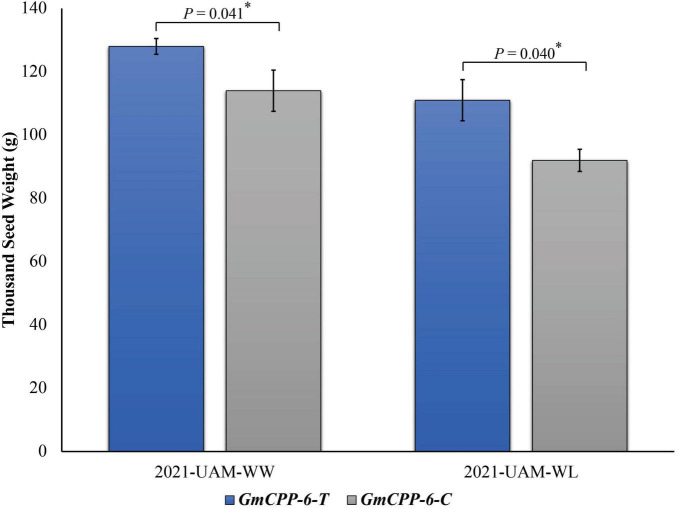
Phenotypic comparison of *GmCPP-6* allelic variations under well water (WW) and water limited (WL) conditions. The two environments were at University of Agriculture Multan (UAM) under WW and WL conditions in year 2021. *p less than 0.05. Error bars denote standard error.

Two allelic variations of *GmCPP-6*, i.e., *GmCPP-6-T* and *GmCPP-6-C* were identified in the studied soybean germplasm. *GmCPP-6-C* was the most frequently occurring allelic variation available in 58.6% of studied soybean accessions. Marker trait association analyses exhibited that at unique field sites, *GmCPP-6-T* was associated with higher thousand seed weight in both environments (Figure 7).

## Discussion

Cysteine-rich polycomb-like protein TFs are quite a small gene family involved in plant growth and stress responses (Andersen et al., 2007; Lu et al., 2013; Zhang et al., 2015; Zhou et al., 2018; Nan et al., 2021). In earlier studies, identification of the *CPP* gene family in *Camellia sinensis*, *A. thaliana*, *C. sativus*, and *G. max* has been performed. But genome-wide characterization, in relation to analyses of sequence polymorphism, has not been performed in soybean. In the present work, a comprehensive identification, characterization, and analysis of sequence polymorphism of *GmCPPs* was performed.

### Soybean cysteine-rich polycomb-like proteins are conserved during evolution

In the present work, we identified 81 *CPP* genes in nine different organisms i.e., chlorophytes (*C. reinhardtii*), lycophytes (*S. moellendorffii*), *Brassicaceae* (*A. thaliana* and *B. rapa*), *Fabaceae* (*G. max, G. soja*, and *C. cajan*), and *Poaceae* (*Z. mays* and *O. sativa*). Phylostratum analyses of *CPPs* indicated that the primitive plant pedigree as *CPPs* were existing in chlorophytes, showing that *CPP* genes originated from early land plants and ortholog genes of *CPP* are existing across kingdom Plantae. Phylogenetic analyses were performed to establish the evolutionary relationship among the studied species. All *CPP* genes were divided into six different clades which showed that most of the *G. max* genes exhibited a close relationship with *G. soja* genes and indicated that both species share a common ancestry. *G. soja* genome has shown to have 0.9154 GB consensus sequences, covering ∼98% of *G. max* genome sequence (Schmutz et al., 2010). Gene structure analyses showed that *GmCPPs* have a higher number of intron which indicates that *GmCPP* belong to the primitive gene family group. Sequence logos for conserved amino acid residues were also highly conserved in *G. max* and *G. soja*. Both N and C terminals of *GmCPPs* and *GsCPPs* are conserved. These results indicate that the *GmCPP* and *GsCPP* genes are evolutionarily conserved which might be helpful to underpin the pattern of *CPP* protein sequence conservation in other members of kingdom Plantae.

### Biophysical characteristics

The estimates of biophysical parameters of all *GmCPP* gene family members delivered helpful information. Biophysical properties predicted that 10 out of 12 *GmCPPs* were positioned in the nucleus. The values of pI and grand average of hydropathicity of all *GmCPPs* indicated that all *CPP* proteins were hydrophilic (<0) and alkaline (∼7) (Supplementary Table 4).

### Exon-intron and motif analyses

The structure of the gene is a vital component that might be contributed by deletion and/or insertion incidents (Lecharny et al., 2003). In past, genome-wide studies have demonstrated that the loss or gain of introns during eukaryotic divergence was widespread (Rogozin et al., 2003; Roy and Penny, 2007). Gene structure analyses showed that all *GmCPP* genes have varied intron lengths that might play crucial roles in the functional divergence of *GmCPP* genes. It has been well-documented that introns play an important role in the evolution of different species (William Roy and Gilbert, 2006). In the current study, we observed that the number of intron for *GmCPPs* ranged from seven to nine indicating that *G. max* has evolved a long time ago (>Million years). Roy and Gilbert also advocated that earlier evolved species have more introns as compared with the newly evolved species (William Roy and Gilbert, 2006). Ten motifs were identified which showed that *CPP* proteins might function in different biological pathways allied with other co-factors. The motif distribution pattern of *CPP* proteins indicated that the distribution was relatively conserved and minimal divergence among the proteins from different groups might be linked with the specific biological function associated with soybean development and stress tolerance.

Transcription is governed by the binding of TFs to promoter *cis*-acting regulatory elements. Various studies have reported the crucial role of *cis*-acting elements in the processes of plant growth and stress responses (Fankhauser and Chory, 1997). In this study, *cis*-acting elements related to plant development and stress responses were identified in the upstream region of *GmCPPs*.

### Gene duplication and selection pressure

The uneven distribution of *GmCPP* genes on chromosomes of *G. max* shows probable gene loss or addition through whole genome or segmental duplication incidents. It has been reported that gene duplication and divergence generally lead toward evolution (Chothia et al., 2003). Gene duplication creates functional differences, which is crucial for speciation and adaptableness in changing environmental conditions (Conant and Wolfe, 2008). Gene duplication indicates that the aligned sequences share > 70% similarity and coverage length > 80% of the entire length (Yang S. et al., 2008). The two duplicated genes present on the different chromosomes of the same sub-genome might be the consequence of segmental or whole genome duplication, whereas, their presence on the same chromosomes might be the consequence of tandem duplication (He et al., 2012). Tandemly duplicated genes tend to be positioned together on chromosomes whereas, in segmental or whole genome duplication, the duplicated genes are generally distributed throughout the genome (Schauser et al., 2005). Approximately 65 million years ago whole genome and segmental duplications in primitive plant species contributed to the expansion of a number of gene families (Barakat et al., 2009; Wang et al., 2013) and contributed genomic complexity to kingdom Plantae (Cannon et al., 2004).

In the present work, we characterized 12 *GmCPP* genes, three times the number of *CPPs* present in chlorophytes, which indicates that *CPP* experienced expansion during their evolution. As reported previously, expansion in the genome permitted many crop plant species to acclimatize to environmental conditions (Ramsey and Schemske, 1998). We noticed that segmental and whole genome duplication were the major reasons responsible for the expansion *CPP* gene family in *G. max*. Segmental type duplication is the main contributor during evolution and it has happened in numerous plant genomes which contain many duplicated chromosomal blocks (Cannon et al., 2004). For instance, many *A. thaliana* gene families experienced evolutionary dynamics that led toward gene family expansion (Baumberger et al., 2003; Wang et al., 2008). Our results showed that *GmCPPs* grouped into pairs (Segmental duplication) which shows an ancient expansion in the gene family in *G. max*. To estimate the selection and environmental pressure, non-synonymous (*Ka*) and synonymous (*Ks*) rates of substitution (*Ka*/*Ks*) were computed. We noticed that *Ka*/*Ks* values of *GmCPP* genes were <1 illustrating that *GmCPP* gene family experienced strong purifying selection pressure.

### Allelic variations influencing seed weight

Soybean PAN-genome might be helpful for bridging the phenotype to genotype gap in soybean breeding. Recently, PAN-genome has been used to discover genes for flowering time in *G. soja* (Li et al., 2014). Marker-assisted selection of elite alleles in breeding programs is vital for ongoing soybean breeding. The utilization of elite allelic variations in cultivars can be enriched if effective molecular platforms are available (Rasheed et al., 2017; Majeed et al., 2018; Ur Rehman et al., 2021). In this study, we used the PAN-genome of *G. max* to explore sequence polymorphism for *GmCPP-6*. Although, sequence polymorphism was explored in all studied *GmCPP* genes the allelic variation was only identified in the CDS region of *GmCPP-6.* Hence, all other genes were excluded for marker-trait association analyses. The absence of polymorphism in all other *GmCPP* genes is possibly due to allele fixation during evolution or because of the lower number of *G. max* accessions available for PAN-genomics studies. More work is required for further confirmation or to investigate these two possibilities. Converting sequence polymorphism to gel-free (KASP) markers enable SNPs to be more efficiently applied in selecting desirable alleles in marker-assisted breeding. Moreover, the KASP assay procedure is cost-effective. In the current study, soybean accessions having *GmCPP-6-T* had higher thousand seed weight under both environmental conditions i.e., WW and WL. Moreover, RNA-Seq Atlas of *G. max* also reported higher expression of *GmCPP-6* in seeds (14–25 days after fertilization) (Severin et al., 2010). Generally, yield-related parameters of crop plants are administered by several genes and are strongly influenced by external stimuli. Pyramiding of favorable alleles might be helpful for continued improvement in soybean. The developed molecular marker will be useful for marker-assisted breeding in soybean which can be used in combination with other molecular markers.

## Conclusion

Eighty-one *CPP* genes were studied in this research, and on the basis of phylogenetic analyses, all genes were divided into six sub-groups. The amino acid residues of *G. max* and *G. soja* demonstrated less conservation in web logos. Introns are present in *GmCPP* genes, and the pattern of protein motif distribution is less consistent across all proteins. Growth regulator *cis*-acting elements were found in the upstream regions of *GmCPP*, indicating their role in plant growth and development. Gene duplication and synteny analysis revealed that the *GmCPP* genes have undergone segmental and whole genome duplication during evolution, resulting in a significant expansion of the *GmCPP*. The current study also delivers molecular marker associated with higher thousand seed weight in soybean. These findings lay the groundwork for further research into the roles of *GmCPP* genes in soybean growth, development, and response to external stimuli.

## Data availability statement

The datasets presented in this study can be found in online repositories. The names of the repository/repositories and accession number (s) can be found in the article/Supplementary material.

## Author contributions

TN, MT, SI, and SU conceived the idea. TN performed the experiment, analyzed the data, and wrote the original draft of the manuscript. MT, SI, MS, GQ, ZK, ZZ, and ZG guided in the execution of field and laboratory experiments. ZK, ZZ, and ZG assisted in the development of molecular markers. MT, SI, AB, and SU reviewed the manuscript. All authors contributed to the article and approved the submitted version.

## Footnotes

1
http://planttfdb.gao-lab.org/link.php

2
http://www.ebi.ac.uk/InterProScan/

3
http://smart.embl-heidelberg.de/

4
http://us.expasy.org/tools/protparam.html

5
http://www.softberry.com/

6
https://mybiosoftware.com/cello-v-2-5-subcellular-localization-predictor.html

7
https://weblogo.berkeley.edu/logo.cgi

8
http://gsds.gao-lab.org/

9
http://memesuite.org

10
https://bar.utoronto.ca/eplant/

11
http://www.lgcgenomics.com

12
https://www.ncbi.nlm.nih.gov/Structure/cdd/wrpsb.cgi

## Supplementary material

Supplementary Figure 1 Showing conserved domain in all *GmCPP* genes. Conserved domain in *GmCPP* genes. Yellow color shows TCR family domains.

Supplementary Table 1 Transcription factor ID, taxonomic ID and predict sub-cellular localization.

Supplementary Table 2 *cis*-acting elements related to plant development, stress, and light responses.

Supplementary Table 3 Gene duplication, collinearity/synteny, and *Ka/Ks* values.

Supplementary Table 4 Biophysical properties of GmCPP.
