# Supplementary figures and images for: Genome-wide characterization and sequence polymorphism analyses of cysteine-rich poly comb-like protein in Glycine max
Source: Front Plant Sci. 2022 Sep 20;13:996265. doi: 10.3389/fpls.2022.996265 (PMC9531024; doi:10.3389/fpls.2022.996265)

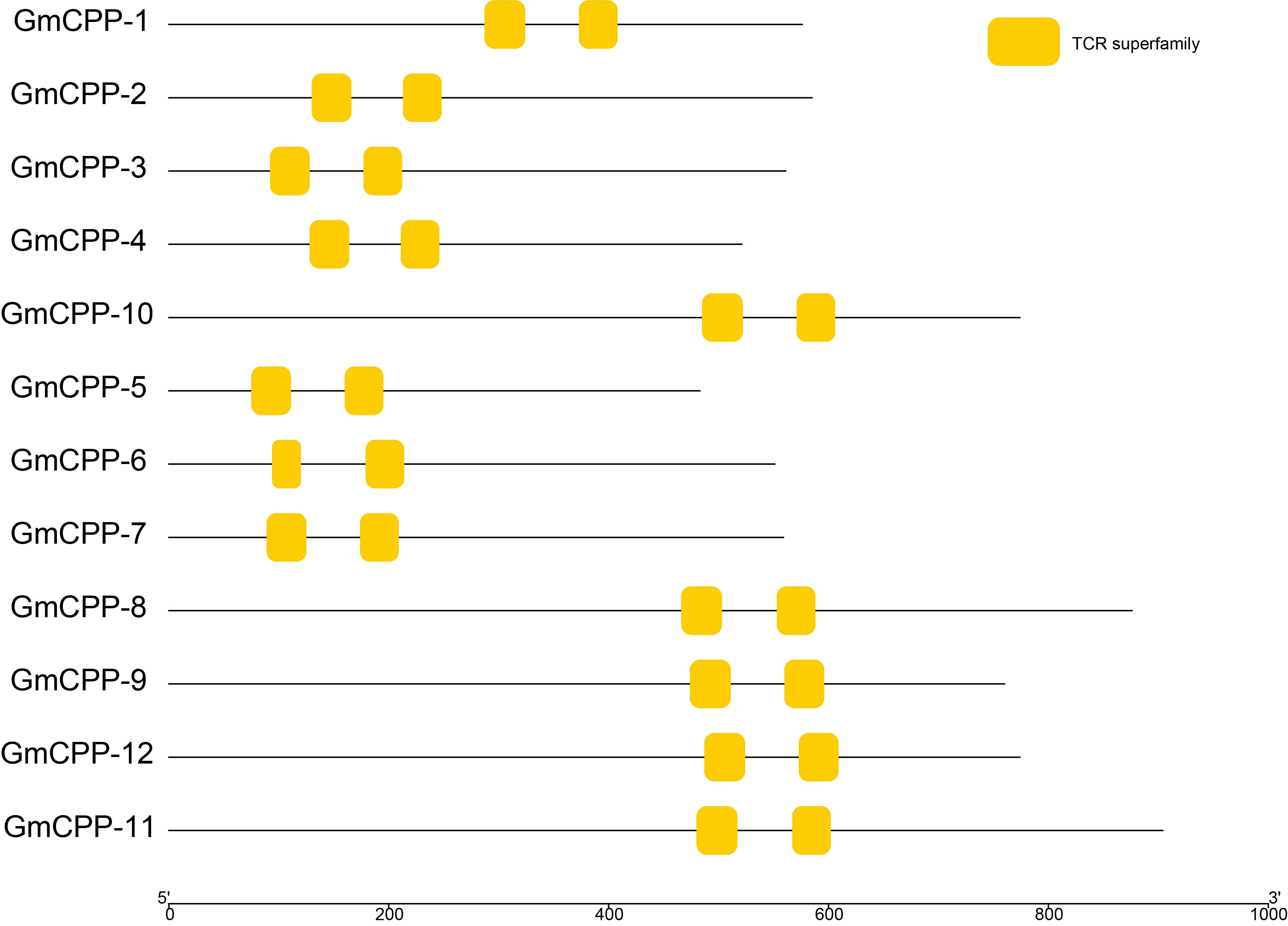

Supplement: Supplementary Figure 1 — Showing conserved domain in all GmCPP genes. Conserved domain in GmCPP genes. Yellow color shows TCR family domains. [file Image_1.JPEG]
